# Supplementary material for: MetaRibo-Seq measures translation in microbiomes
Source: Nat Commun. 2020 Jun 29;11:3268. doi: 10.1038/s41467-020-17081-z (PMC7324362; doi:10.1038/s41467-020-17081-z)
Supplement: Supplementary file 10 — Supplementary Data 7 [file 41467_2020_17081_MOESM10_ESM.zip › File2/Confidence_High_Taxonomy/158737_out.krona.html]

Javascript must be enabled to view this page.

members
magnitude
magnitudeUnassigned
count
unassigned
taxon
rank

158737\_out

12

12
superkingdom
2

12
phylum
1239

12
class
186801

186802
order
12

12
family
541000

12

SRS015578\_contig\_number\_contig-100\_7940.7940SRS015854\_contig\_number\_contig-100\_27285.60488SRS043001\_contig\_number\_contig-100\_13367.13367SRS148874\_contig\_number\_contig-100\_21969.21970
4
1263
genus

2292375

SRS105153\_contig\_number\_2827
1
species

1638786

SRS014979\_contig\_number\_36878SRS104311\_contig\_number\_contig-100\_1003.238546SRS144183\_contig\_number\_22393
3
species

species
4

SRS015431\_contig\_number\_4372SRS019068\_contig\_number\_83089SRS050752\_contig\_number\_16697SRS971275\_contig\_number\_contig-100\_1511.279170
40519
